# Differentiation of hepatocellular carcinoma from hepatic hemangioma using diffusion-derived vessel density map-based radiomics features

**DOI:** 10.3389/fonc.2026.1828632

**Published:** 2026-05-28

**Authors:** Jingwen Yu, Lijian Liu, Long Qian, Wenming Deng, Jujian Guo, Yijia Zheng, Dehong Luo, Zhou Liu

**Affiliations:** 1 Department of Radiology, National Cancer Center/National Clinical Research Center for Cancer/Cancer Hospital & Shenzhen Hospital, Chinese Academy of Medical Sciences and Peking Union Medical College, Shenzhen, Guangdong, China; 2 Department of Biomedical Engineering, College of Engineering, Peking University, Beijing, China

**Keywords:** diffusion-derived vessel density, diffusion-weighted imaging, hepatic hemangioma, hepatocellular carcinoma, radiomics

## Abstract

**Objective:**

To evaluate the diagnostic performance of radiomics features extracted from diffusion-derived Vessel Density (DDVD) in differentiating hepatocellular carcinoma (HCC) from hepatic hemangioma (HG).

**Methods:**

This retrospective study enrolled 232 patients (104 with pathologically confirmed HCCs and 128 with clinically diagnosed HGs). The cohort was randomly divided into training and testing sets (7:3 ratio). We generated DDVD maps (subtraction maps of b0-b50 voxel-by-voxel). Features were extracted from b0, b50, b800, ADC, and DDVD maps, respectively. Feature selection was sequentially performed for each type of image using Mann-Whitney U test, Pearson correlation (*|r*| > 0.8), and LASSO regression. Five Logistic regression models (b0, b50, b800, ADC, and DDVD) were independently constructed to differentiate HCC from HG, with model performance evaluated using receiver operating characteristic analysis, with AUC, sensitivity, specificity, NPV, PPV, and accuracy as primary metrics. Delong test was utilized to evaluate the difference in performance of models.

**Results:**

From a total of 1,197 features initially extracted, 10 most informative features from each image type were retained. The DDVD-based model demonstrated comparable performance to b0, b50, and ADC models, achieving AUC values of 0.926 (95% CI: 0.914 - 0.929) in the validation cohort and 0.977 (95% CI: 0.948 - 1.000) in the independent test cohort. Comparative analysis revealed that the b0, b50, ADC, and DDVD models significantly outperformed the b800 model in the test cohort (all *p* < 0.05).

**Conclusions:**

DDVD-based radiomics demonstrates an effective approach for differentiating HCC from HG by quantifying spatial heterogeneity in microvascular characteristics.

## 1 Introduction

Hepatocellular carcinoma (HCC) is distinguished as one of the most vascularized solid tumors, where angiogenesis is pivotal for its development, progression, and metastasis (1, 2). In contrast, hepatic hemangioma (HG) is the common benign vascular lesion in the liver (3, 4). Early detection and accurate diagnosis of these two lesions are extremely important because their treatment strategies and prognoses differ completely. HCC typically requires immediate intervention, including surgical resection, liver transplantation, or interventional therapy, whereas HG generally does not need special treatment and necessitates intervention only when it reaches a significant size or elicits symptoms (5, 6). Although HCC and HG are less challenging to differentiate for the cases with typical findings, they could share similar imaging findings in the early stages or for atypical cases. The gold standard for differential diagnosis involves pathological evidence, mainly by needle biopsy (7). However, needle biopsy is an invasive examination that may lead to complications, such as needle tract seeding for HCC, bleeding for HCC and HG. Particularly for HG, which is composed of numerous abnormally dilated vascular lacunae filled with blood, the puncture might easily rupture the vascular wall of the tumor, leading to uncontrollable bleeding and intratumoral hematoma (8), which has made puncture biopsy a somewhat contraindication for HG in clinical practice. Furthermore, a negative biopsy does not rule out the possibility of HCC. It has been reported that the false negative rate of biopsy can reach as high as 30% (9). Consequently, a non-invasive approach allowing for precise differential diagnosis is highly desirable for tailoring appropriate treatment strategies.

MRI has recently gained prominence in the diagnosis of hepatic tumors because of its various modalities such as multi-parametric imaging, functional imaging, and biochemical metabolic analysis techniques, all of which can optimize the clinical application from morphology to quantitative analysis (10). Previous studies have achieved promising performance in differentiating HCC from HG by extracting radiomics features from conventional sequences [in-phase T1WI, out-phase T1WI, T2WI, and diffusion-weighted imaging (DWI)] scan images (11).

Recently, Wang et al. introduced a DWI-derived biomarker, diffusion-derived vessel density (DDVD), to assess the micro-perfusion of liver tissue. This newly introduced approach reflects the volume of microvessels per tissue unit volume and reveals the extent of microperfusion inside a local tissue (12). When the MRI diffusion gradient is off (ie, b = 0 s/mm2), the vessels with flowing blood show high signals; when the diffusion gradient is on using a nonzero low b-value, vessels with flowing blood appear as signal void. Thus, the signal difference between gradient-off (b = 0) and gradient-on low-b-value images can reflect regional microvascular density, allowing the derivation of a quantitative biomarker for physiological vascular perfusion (13–15). From the perspective of intravoxel incoherent motion (IVIM) theory, the ultra-low b-value range of 0–50 s/mm² is predominantly sensitive to microperfusion induced by intravascular blood flow, rather than slow water diffusion in liver parenchyma or T2 relaxation effects. Within this low b-value interval, diffusion-weighted signal attenuation is mainly dominated by incoherent microvascular blood flow, whereas tissue water diffusion and T2-related contrast remain stable and exert minimal confounding effects (13, 16, 17). Accordingly, pixel-wise signal subtraction between b = 0 and b = 50 s/mm² can specifically isolate microvascular perfusion components and reliably reflect tissue vascular density. Given that both HCC and HG are highly vascularized lesions yet demonstrate distinct enhancement patterns, we hypothesize that DDVD may have diagnostic value in discriminating HCC from HG. Hence, in this study, we investigated the utility of DDVD for discriminating HCC from HG and compared the discriminatory value of high-throughput radiomics features derived from conventional DWI sequences versus DDVD.

## 2 Materials and methods

### 2.1 Patients

**Figure 1:**
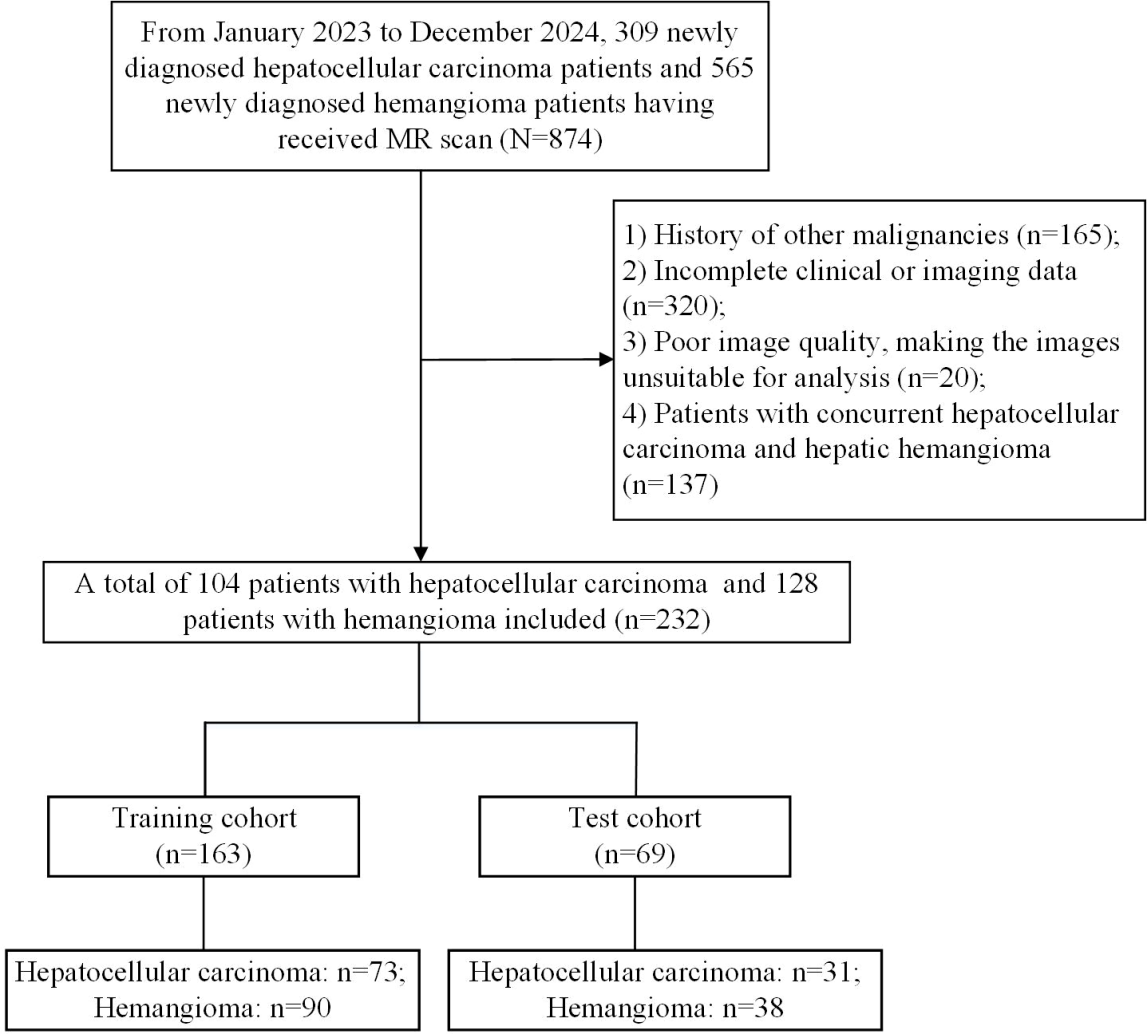
Flowchart of the patient enrollment process and study cohorts.

This retrospective study was approved by the Ethics Committee of our institution, and informed consent from each subject was waived (ethics number: KYLX2021-38). From January 2023 to December 2024, 104 patients with pathologically confirmed HCC and 128 patients with clinically confirmed HG (without pathological confirmation) were enrolled. Inclusion criteria were: (1) Pathologically diagnosed HCC or clinically confirmed HG; (2) Abdominal MRI protocol with available DWI sequences with b = 0, b = 50, b = 800, and ADC maps available; (3) No prior treatments such as biopsy, radiotherapy, chemotherapy, or resection before the MRI examination. Exclusion Criteria: (1) History of other malignancies; (2) Incomplete clinical or imaging data; (3) Poor image quality making the images unsuitable for analysis; (4) Patients with concurrent HCC and HG. A total of 232 patients were randomly divided into a training cohort (N = 163) and a test cohort (N = 69) in a ratio of 7:3 by using a completely random classification method. **Figure 1** summarizes the enrollment of the study cohorts.

### 2.2 MR data acquisition

Magnetic Resonance (MR) examinations were conducted using a 1.5 Tesla MR scanner (Aera, Siemens Healthineers, Erlangen, Germany) equipped with a 16-channel body matrix coil. The diffusion imaging was based on a single-shot spin-echo–type echo-planar sequence. Three-motion probing gradient directions were applied for each b-value. The spectral attenuated inversion recovery (SPAIR) technique was used for fat suppression. Diffusion images with three b-values of 0, 50, and 800 s/mm^2^ were acquired. Imaging parameters included a TR of 4300 ms, TE of 57 ms, slice thickness of 5 mm, interslice gap of 1 mm, field of view (FOV) of 340 mm × 340 mm, number of excitations (NEX) of 1, 2, and 6, respectively, and 32 slices.

### 2.3 Image preparation and tumor segmentation

**Figure 2:**
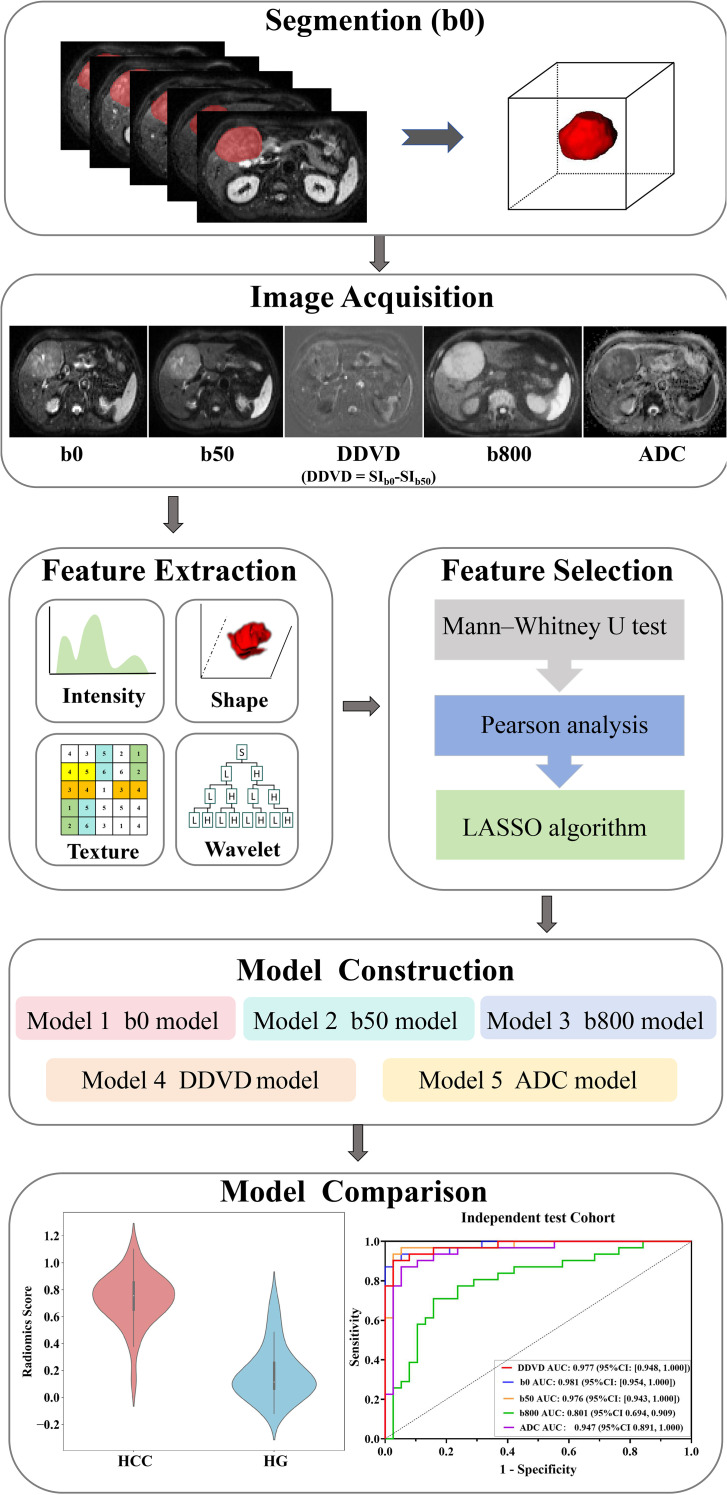
The workflow of the whole study.

We implemented the pixel-wise subtraction of b0 and b50 images to generate a difference image (DDVD), thereby extracting the differential information between the two images, with the calculation formula: DDVD = signal intensity of b0 map - signal intensity of b50 map. A radiologist (L.J.L.) with more than a decade of experience in hepatobiliary imaging reviewed the images and delineated the regions of interest (ROI) on b0 images using the ITK-SNAP software (www.itksnap.org). Lesion location and size were initially determined using standard T2-weighted and T1-weighted gadolinium-enhanced images. In the case of multiple lesions, only the largest lesion was chosen. The same ROI is also applied to b50, DDVD, b800, and ADC images. **Figure 2** presents the flowchart illustrating the entire research design.

### 2.4 Radiomics feature extraction

Before radiomics feature extraction, all images underwent preprocessing, including resampling and standardization, to ensure result repeatability. Radiomics features for each ROI were extracted using Pyradiomics (https://pypi.org/project/pyradiomics/), following the Image Biomarker Standardisation Initiative (IBSI) guidelines (18). A total of 1197 features were extracted for each 3D lesion of each patient. These features included first-order intensity, shape, texture, and filtered features (**Supplementary S1**).

### 2.5 Feature selection

The z-score normalization technique was utilized to standardize all features. Firstly, Mann-Whitney U test was used to select the features with significant differences (*p* < 0.05) between HCC and HG groups. Then, Pearson’s correlation coefficient was computed to assess the correlation between each two of the initially selected features. When the correlation coefficient between any two features exceeded 0.8, the feature demonstrating a smaller P-value in the Mann-Whitney U test was retained. The top 10 radiomics features with the largest absolute coefficients in the training cohort were identified and selected using the least absolute shrinkage and selection operator (LASSO) method, and the optimal value of the parameter λ was determined based on a 10-fold cross-validation. Individual radiomics scores (Rad-scores) were calculated from a linear combination of each feature along with its coefficient weighting according to the non-zero coefficient features selected by LASSO. The scikit-learn package in Python was utilized for LASSO regression modeling.

### 2.6 Development of radiomics signature model

After performing LASSO feature screening, the Logistic regression classifier was trained using the selected radiomics features. Firstly, the training set was iteratively and randomly split into training and validation subsets at a 7:3 ratio, with this partitioning procedure repeated 100 times to evaluate the variability of our model. Then, the model was validated on an independent test cohort. The diagnostic performance of the models was assessed using various metrics, including areas under the receiver operating characteristic (ROC) curve (AUC), specificity, sensitivity, negative predictive value (NPV), positive predictive value (PPV), and accuracy.

## 3 Results

### 3.1 Patient characteristics

Of the 232 patients included in the study, 156 were males and 76 were females. The mean age of patients is 53.50 ± 12.85 years (range, 23–83 years). The clinical characteristics of the training and test cohorts were summarized in **Table 1**. No significant difference was found between the two cohorts in any relevant clinical risk factors. There were significant differences in age, long-axis diameter and short-axis diameter of lesions, as well as the prevalence of underlying liver cirrhosis and hepatitis B between the HCC and HG groups in both the training and test cohorts (all *p* < 0.05). Sex and the prevalence of hepatitis C differed significantly between the two groups only in the training cohort (*p* < 0.05). Baseline patient characteristics are summarized in **Table 2**.

**Table 1:** Comparison of patient characteristics among different cohorts.

Characteristic	Training cohort (N = 163)	Test cohort (N = 69)	*p* value
Age			
< 45	40 (24.54)	24 (34.78)	0.11
≥ 45	123 (75.46)	45 (65.22)	
Gender			
M	107 (65.64)	49 (71.01)	0.43
F	56 (34.36)	20 (28.99)	
Lesion long-axis diameter (cm)	3.50 (2, 6.90)	3.80 (2.05, 5.90)	0.99
Lesion short-axis diameter (cm)	2.70 (1.60, 5.40)	2.60 (1.60, 5)	0.87
Presence of liver cirrhosis	52 (31.90)	22 (31.88)	0.998
Presence of hepatitis B infection	57 (34.97)	30 (43.48)	0.221
Presence of hepatitis C infection	9 (5.52)	1 (1.45)	0.163

Normally distributed continuous variables are presented as mean ± SD (minimum value, maximum value) and non-normally distributed continuous variables are presented as median (interquartile range). Categorical variables are expressed as n (%).

**Table 2:** Baseline characteristics of patients in the training and test cohorts.

Characteristic	Training cohort (N = 163)			Test cohort (N = 69)		
	HCC (n = 73)	HG (n = 90)	*p* value	HCC (n = 31)	HG (n = 38)	*p* value
Age						
< 45	6 (8.22)	34 (37.78)	< 0.001	6 (19.35)	18 (47.37)	0.02
≥ 45	67 (91.78)	56 (62.22)		25 (80.65)	20 (52.63)	
Sex						
M	63 (86.30)	44 (48.89)	< 0.001	25 (80.65)	24 (63.16)	0.11
F	10 (13.70)	46 (51.11)		6 (19.35)	14 (36.84)	
Lesion long-axis diameter (cm)	5.30 (3.35, 9.95)	2.40 (1.40, 4.03)	< 0.001	5.30 (3.10, 8.90)	2.65 (1.48, 4.33)	< 0.001
Lesion short-axis diameter (cm)	4.50 (2.80, 7.10)	1.80 (1.10, 3.23)	< 0.001	4.50 (2.60, 7.10)	2.00 (1.10, 3.03)	< 0.001
Presence of liver cirrhosis	52 (71.23)	0	< 0.001	21 (67.74)	1 (2.63)	< 0.001
Presence of hepatitis B infection	52 (71.23)	5 (5.56)	< 0.001	26 (83.87)	4 (10.53)	< 0.001
Presence of hepatitis C infection	9 (12.33)	0	0.001	1 (3.23)	0	0.265

Normally distributed continuous variables are presented as mean ± SD (minimum value, maximum value) and non-normally distributed continuous variables are presented as median (interquartile range). Categorical variables are expressed as n (%).

### 3.2 Radiomics feature selection and radiomics score calculation

**Figure 3:**
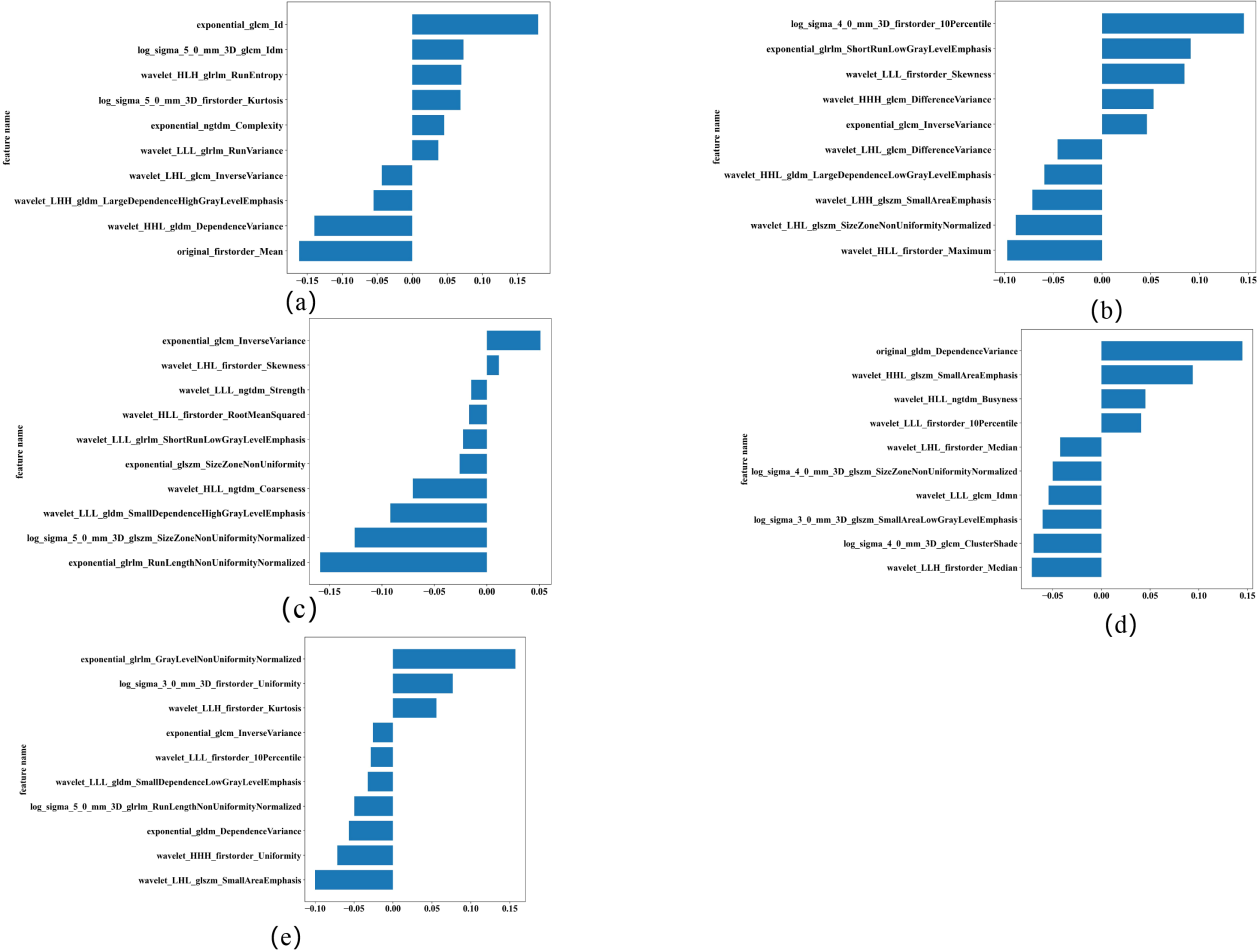
The optimal 10 features with the largest absolute coefficients subsets selected by LASSO and their correlation coefficients. **(a)** ADC, **(b)** DWI b0, **(c)** DWI b50, **(d)** DWI b800, **(e)** DDVD.

**Figure 4:**
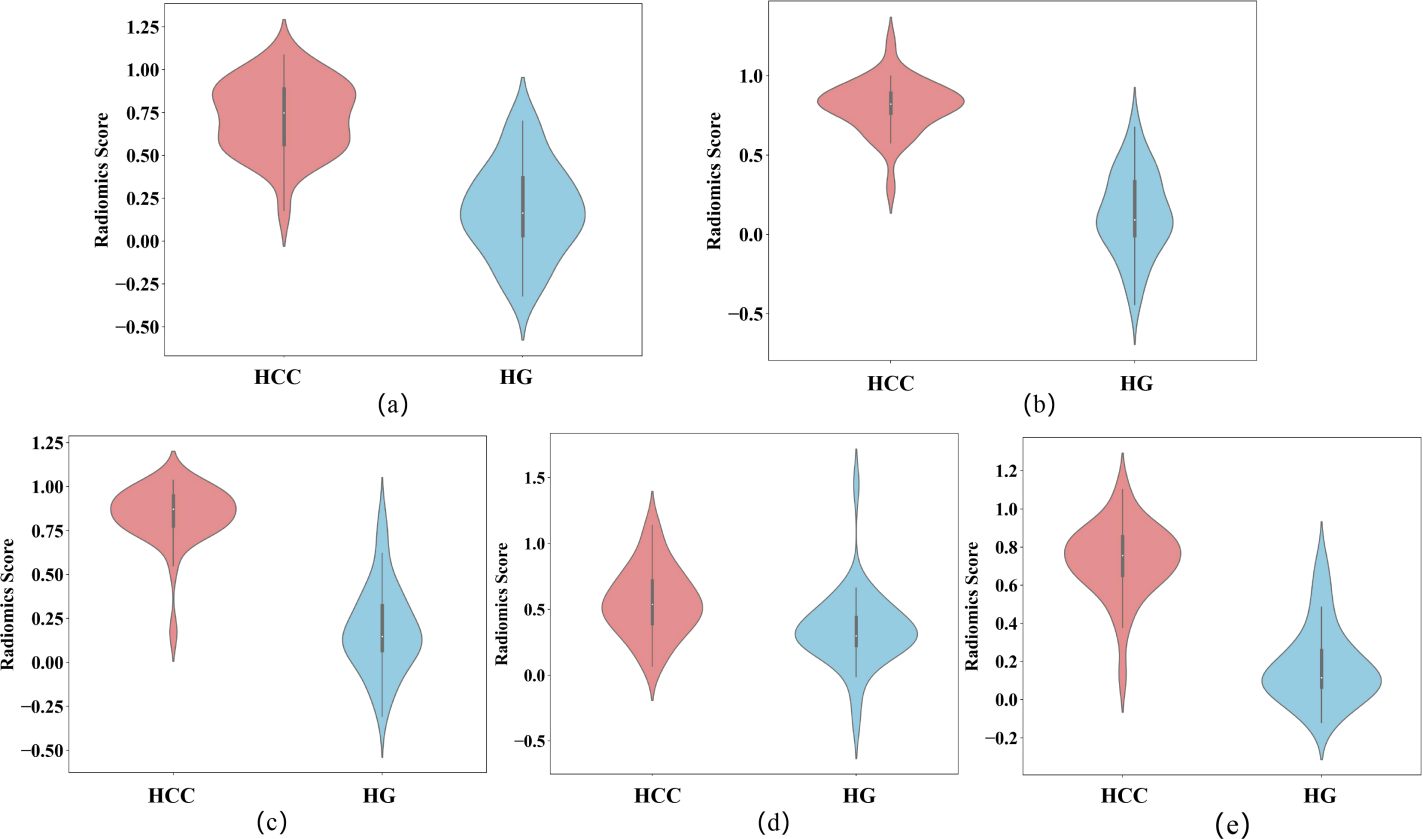
Violin plot visualization comparing the distribution of radiomics scores between HCC and HG in the test cohort. **(a)** ADC, **(b)** DWI b0, **(c)** DWI b50, **(d)** DWI b800, **(e)** DDVD.

**Figure 5:**
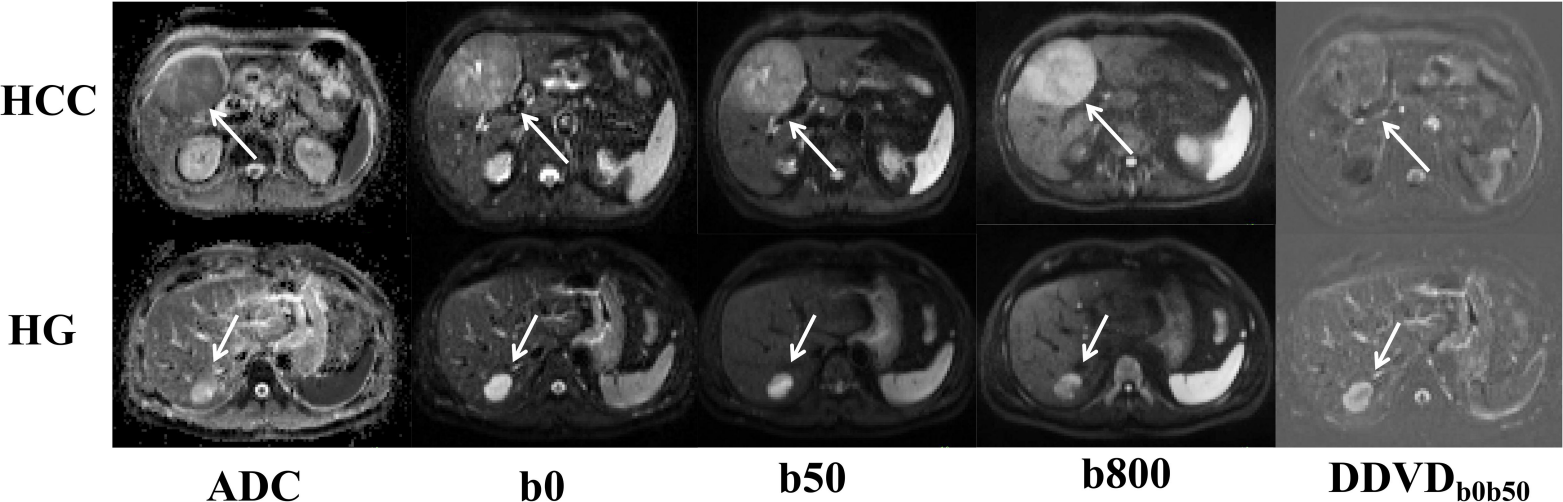
A 68-year-old man with hepatocellular carcinoma (HCC). Radiomics scores (Rad-score): 0.51 (ADC), 0.81 (b0), 0.96 (b50), 1.14 (b800), 0.78 (DDVD). A 56-year-old man with hemangioma (HG). Rad-score: 0.06 (ADC), 0.05 (b0), 0.39 (b50), 0.44 (b800), 0.11(DDVD).

After the Mann-Whitney U test, 1066, 1070, 995, 849, and 1034 features were selected for b0, b50, DDVD, B800, and ADC map, respectively. Subsequently, Pearson correlation analysis was conducted, resulting in the following number of retained features for each map: b0 (n = 108), b50 (n = 103), DDVD (n = 98), b800 (n = 128), ADC (n = 100). Finally, the LASSO analysis selected the optimal 10 features with the largest absolute coefficients from these remaining features. **Figure 3** displays the selected features and their corresponding coefficients used to calculate the Rad-score for each patient, while **Supplementary S2** illustrates the correlation distribution among the selected features. In the test cohorts, we compared the Rad-scores of the HCC group and the HG group for each type of image. The significantly higher Rad-score in the HCC group than in that of the HG group was shown for all types of images (**Figures 4**, **5**).

### 3.3 Model evaluation

**Figure 6:**
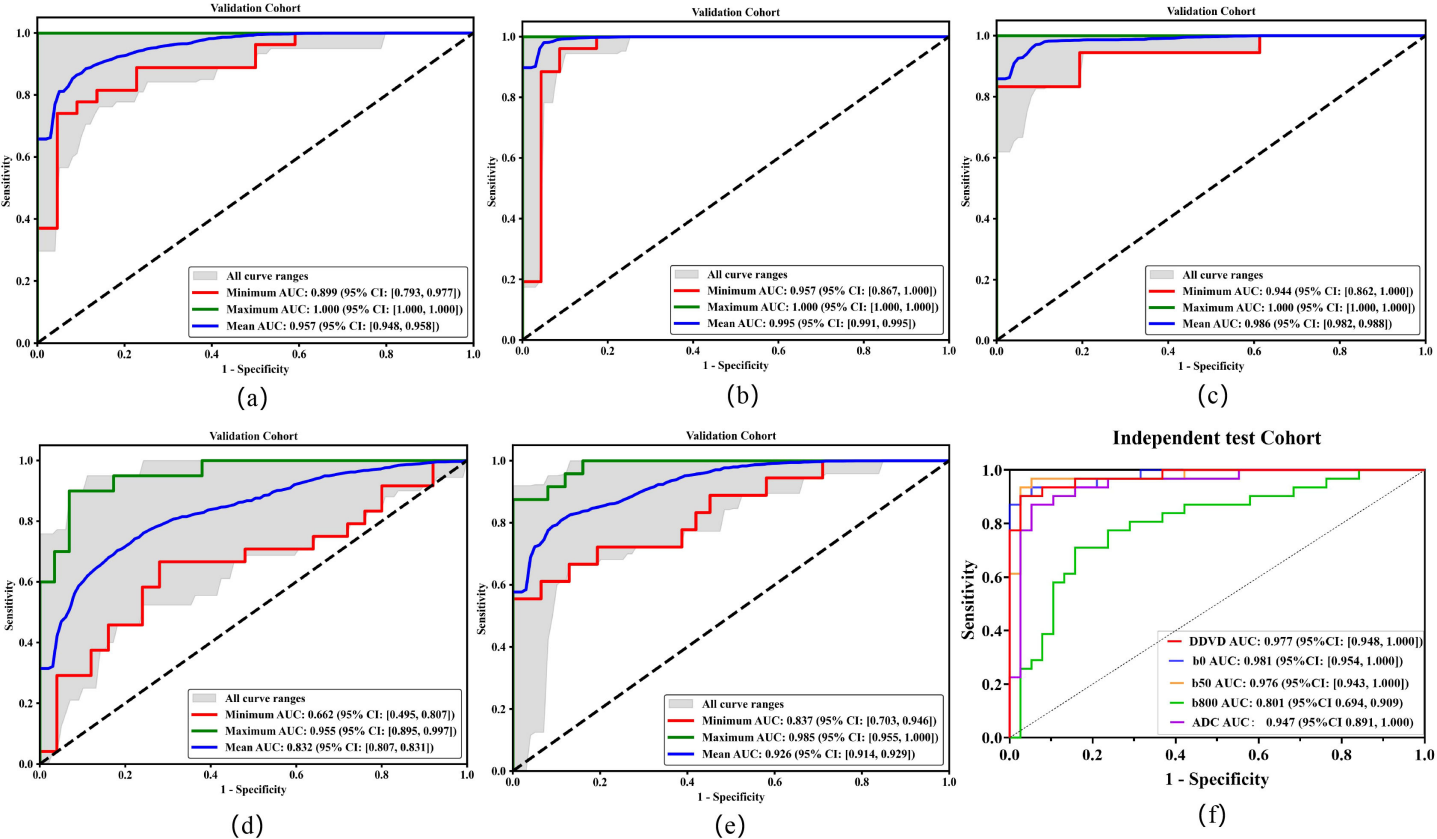
Comparison of receiver operating characteristic curves for each map on the validation set **(a–e)** and the independent test set **(f)**. **(a)** ADC, **(b)** DWI b0, **(c)** DWI b50, **(d)** DWI b800, and **(e)** DDVD.

The prediction performances of the radiomics models are presented in **Table 3** and **Figure 6**. The DeLong test was used to compare the performances across different models in the independent cohort. The study results found that the model performance of the b800 map was significantly lower than the models based on the b0, b50, ADC, and DDVD maps (b0: *p* = 0.001; b50: *p* < 0.001; DDVD: *p* = 0.001; ADC: *p* = 0.011). However, pairwise comparisons revealed no statistically significant differences between any two of the b0, b50, DDVD, and ADC models (all *p* > 0.05), with *p*-values of 0.189 (ADC vs. b0), 0.255 (ADC vs. b50), 0.227 (ADC vs. DDVD), 0.755 (b0 vs. b50), 0.774 (b0 vs. DDVD), and 0.953 (b50 vs. DDVD).

**Table 3:** Performance of ADC, b0, b50, b800, and DDVD models in differentiation of HCC and HG.

Model	AUC	Sensitivity	Specificity	PPV	NPV	Accuracy
ADC						
Validation Cohort	0.957 (0.948-0.958)	0.903 (0.891-0.915)	0.924 (0.913-0.936)	0.911 (0.898-0.924)	0.921 (0.910-0.931)	0.915 (0.907-0.923)
Independent test Cohort	0.947 (0.891-1)	0.871 (0.753-0.989)	0.947 (0.876-1)	0.931 (0.839-1)	0.9 (0.807-0.993)	0.913 (0.847-0.98)
b0						
Validation Cohort	0.995 (0.991-0.995)	0.985 (0.980-0.990)	0.984 (0.980-0.989)	0.981 (0.975-0.987)	0.987 (0.983-0.991)	0.984 (0.981-0.988)
Independent test Cohort	0.980 (0.952-1)	0.935 (0.849-1)	0.947 (0.876-1)	0.935 (0.849-1)	0.947 (0.876-1)	0.942 (0.887-0.997)
b50						
Validation Cohort	0.986 (0.982-0.988)	0.976 (0.970-0.983)	0.953 (0.945-0.961)	0.944 (0.935-0.954)	0.980 (0.975-0.985)	0.963 (0.959-0.968)
Independent test Cohort	0.976 (0.943-1)	0.968 (0.906-1)	0.947 (0.876-1)	0.938 (0.854-1)	0.973 (0.921-1)	0.957 (0.908-1)
b800						
Validation Cohort	0.832 (0.807-0.831)	0.752 (0.728-0.776)	0.844 (0.828-0.860)	0.808 (0.791-0.824)	0.808 (0.792-0.824)	0.802 (0.792-0.812)
Independent test Cohort	0.801 (0.694- 0.909)	0.71 (0.55-0.869)	0.842 (0.726-0.958)	0.786 (0.634-0.938)	0.78 (0.654-0.907)	0.783 (0.685-0.88)
DDVD						
Validation Cohort	0.926 (0.914-0.929)	0.840 (0.822-0.858)	0.916 (0.900-0.931)	0.900 (0.884-0.915)	0.878 (0.865-0.890)	0.881 (0.874-0.888)
Independent test Cohort	0.977 (0.948-1)	0.903 (0.799-1)	0.974 (0.923-1)	0.966 (0.899-1)	0.925 (0.843-1)	0.942 (0.887-0.997)

## 4 Discussion

In this study, we employed radiomics methodology to investigate the diagnostic efficacy of DDVD images in differentiating HCC from HG, with comparative analysis against conventional b0, b50, b800, and ADC maps. The results demonstrated that DDVD-based radiomics model achieved a comparable performance in the differentiation with models based on the conventional b0, b50, and ADC maps (*p* > 0.05), but a significantly better performance than that of the b800 model.

DDVD, as an emerging biological marker, was initially employed by Wang et al. for the assessment of liver fibrosis and HCC perfusion status (14, 19). Subsequently, it has been further utilized to differentiate between normal pregnancies and those with early-onset preeclampsia, as well as to evaluate placental perfusion, among other applications (20, 21). These advancements underscore DDVD’s potential as a multi-organ perfusion biomarker. However, a significant limitation common to previous DDVD-based studies is their neglect of the spatial heterogeneity in vasculature density within tissues. Building on this foundation and the work of Wang et al., the present study innovatively integrates radiomics methodology into the analysis of DDVD maps. HCC and HG possess distinct vascular and hemodynamic characteristics, which provide a pathophysiological basis for using DDVD-based radiomics analysis to differentiate HCC and HG. HCC is a highly vascularized tumor characterized by abnormal neovascularization and disorganized vascular networks. These newly formed vessels typically exhibit increased permeability and irregular vessel walls, resulting in elevated microvessel density but distinct hemodynamic characteristics compared to normal liver tissue (22). In contrast, HG, a benign vascular lesion, is composed of numerous dilated vascular channels with thicker and more regular vessel walls. Although HG also demonstrates high vascular density, its hemodynamic features differ significantly from HCC, typically manifesting as slow blood flow and reduced microvascular permeability (23, 24). Through the subtraction of b50 images from b0 images, we generated a novel composite image that indirectly reflects tissue microvascular density. We further extracted additional quantitative features to quantify the spatial heterogeneity from these images using radiomics analysis. Accordingly, our result showed that the DDVD-based radiomics model demonstrates comparable performance with models based on the conventional b0, b50, and ADC maps.

Previously, Wu et al. utilized a DWI sequence with b=600 s/mm² to differentiate HCC from HG with a suboptimal AUC of 0.73 for the random forest model and 0.65 for the logistic regression model (11), which is lower than that of the b800 model in our study (AUC = 0.801). Our studies differed in the sample size, inclusion criteria, b value, feature selection method, etc., which need further confirmation in the future.

In the present study, three b-values (0, 50, and 800 s/mm2) were used to create the ADC maps. In Nam’s study, ADC maps were constructed using b50 and b800. The results demonstrated excellent diagnostic accuracy of quantitatively measured ADC values in differentiating HCC from HG, with an AUC of 0.995 (95% CI: 0.969-1.000) (25). Consistently, in our study, the weighting coefficient for original_firstorder_mean had the second-highest absolute value among all features, indicating that the mean ADC value is a significant feature in distinguishing between these two lesions.

Although the b0, b50, and b800 images are acquired during a single DWI scan sequence using diffusion gradients with distinct b-values, they encode divergent tissue information. The b0 image (b=0 s/mm²) lacks diffusion gradients, and its signal intensity primarily reflects T2 relaxation properties, visualizing hepatic parenchymal T2 contrast and macrovascular flow (26, 27). The b50 image employs a low b-value, exhibiting high sensitivity to microcirculatory perfusion. The b800 image applies a high b-value, where signal attenuation is dominated by restricted water diffusion, with negligible contributions from T2 effects or perfusion. As illustrated in **Figure 5**, T2-weighted imaging characteristics result in a “light bulb sign” with homogeneous signal intensity in HG on both DWI b0 and b50 maps (28). This homogeneity likely stems from HG’s regular cellular arrangement and consistent extracellular space. Conversely, HCC exhibits heterogeneous signal intensity, attributable to pathological alterations such as internal necrosis, which create substantial variations in the water molecule diffusion environment (29). At a b-value of 800, the T2 effect is further suppressed. Under these conditions, the densely packed tumor cells and relatively uniform extracellular space of HCC lead to uniformly expressed water molecule diffusion restriction, resulting in a more homogeneous signal pattern (30). In contrast, the inherent heterogeneity of HG components—including regional differences in cell proliferation activity and extracellular matrix composition—causes varying degrees of water molecule diffusion restriction, manifesting as signal heterogeneity on b800 images. This convergence in diffusion restriction expression contributes to the greater overlap observed in the Rad-scores between HCC and HG on the b800 violin plot. The ADC map, derived computationally, quantifies water diffusivity. It eliminates T2 shine-through effects and mitigates susceptibility artifacts (31, 32). DDVD map reflects tissue microvascular density (33). Our study demonstrated that radiomics models constructed using b0, b50, ADC, and DDVD maps achieved significantly superior diagnostic efficacy in differentiating HCC from HG compared to the b800-based model (*p* < 0.05). This finding warrants further validation through prospective multicenter studies.

Although DDVD showed no statistically significant diagnostic superiority over conventional b0, b50 and ADC maps, its post-processing workflow is still reasonable. Different from conventional DWI which only reflects grayscale signal intensity, DDVD provides an independent quantitative dimension to evaluate lesion microvascular density and microperfusion based on IVIM biophysical mechanism. It improves inter-scan and inter-patient standardization, reduces the interference of T2 shine-through effect, and offers a novel non-invasive perspective for vascular evaluation and differential diagnosis of HCC and HG.

Our study has the following limitations. Firstly, this research is a single-center, small-sample retrospective study with inherent baseline differences in age, lesion size, and background liver disease between HCC and HG groups. In addition, some detailed etiological data such as alcoholic liver disease and MAFLD were incompletely recorded in retrospective medical records; therefore, future multi-center, large-sample prospective studies are needed to validate our findings. Secondly, while b-values of 0 and 50 s/mm² have been conventionally employed for DDVD calculation, systematic investigation of alternative lower b-value combinations (e.g., b=1, 2, 10, 20 s/mm²) is warranted to optimize the sensitivity and specificity of DDVD measurements. Thirdly, the inclusion of HG relied on clinical assessment without histopathological confirmation, inherently favoring larger lesions due to the exclusion of atypical HGs (typically size-stable without pathological confirmation). This selection bias toward clinically apparent HGs may overestimate diagnostic accuracy in our model, particularly limiting its applicability to small or atypical lesions.

## 5 Conclusions

DDVD-based radiomics, by quantifying spatial heterogeneity in tissue microvascular characteristics, provides an effective approach for differentiating HCC from HG.

## Data Availability

The datasets used during the current study are available from the corresponding author on reasonable request. Requests to access the datasets should be directed to ZL, zhou_liu8891@yeah.net.
