# Supplementary material for: Differentiation of hepatocellular carcinoma from hepatic hemangioma using diffusion-derived vessel density map-based radiomics features
Source: Front Oncol. 2026 May 28;16:1828632. doi: 10.3389/fonc.2026.1828632 (PMC13253247; doi:10.3389/fonc.2026.1828632)
Supplement: Supplementary file 1 [file DataSheet1.docx]

Supplementary Material

# Supplementary Table

**Table S1: The extracted features are categorized into seven distinct groups**

| Features | Number |
| --- | --- |
| shape | n = 14 |
| first-order statistics | n = 18 |
| grey level co-occurrence matrix (GLCM) | n = 22 |
| grey level run length matrix (GLRLM) | n =16 |
| grey level size zone matrix (GLSZM) | n = 16 |
| neighboring gray tone difference matrix (NGTDM) | n = 5 |
| gray level dependence matrix (GLDM) | n = 14 |
| filtered features* | |
| exponential transform | n = 91 |
| laplacian of gaussia (sigma: [3.0, 4.0, 5.0]) | n = 273 |
| wavele (-LHL, LHH, HLL, LLH, HLH, HHH, HHL, LLL) | n = 728 |

*Twelve filters were applied to the original images, yielding derived images for each patient.All categories of features except shape features were recalculated on the derived images.

# Supplementary Figures

**Figure S1:** **Correlation heatmaps of the optimal 10 least absolute shrinkage and selection operator (LASSO)-selected predictive feature subsets. (a) ADC. (b) DWI b0. (c) DWI b50. (d) DWI b800. (e) Diffusion-derived vessel density**

**
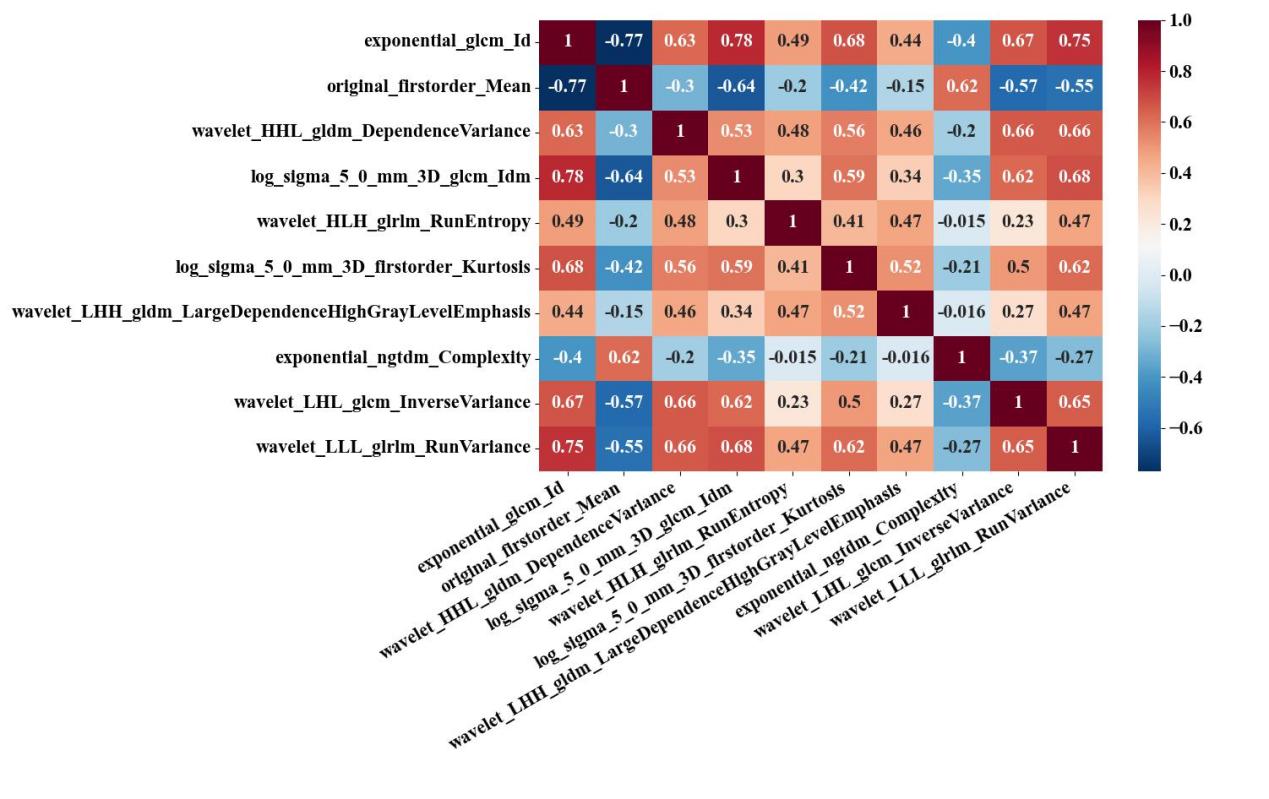
**

1. **ADC**

**
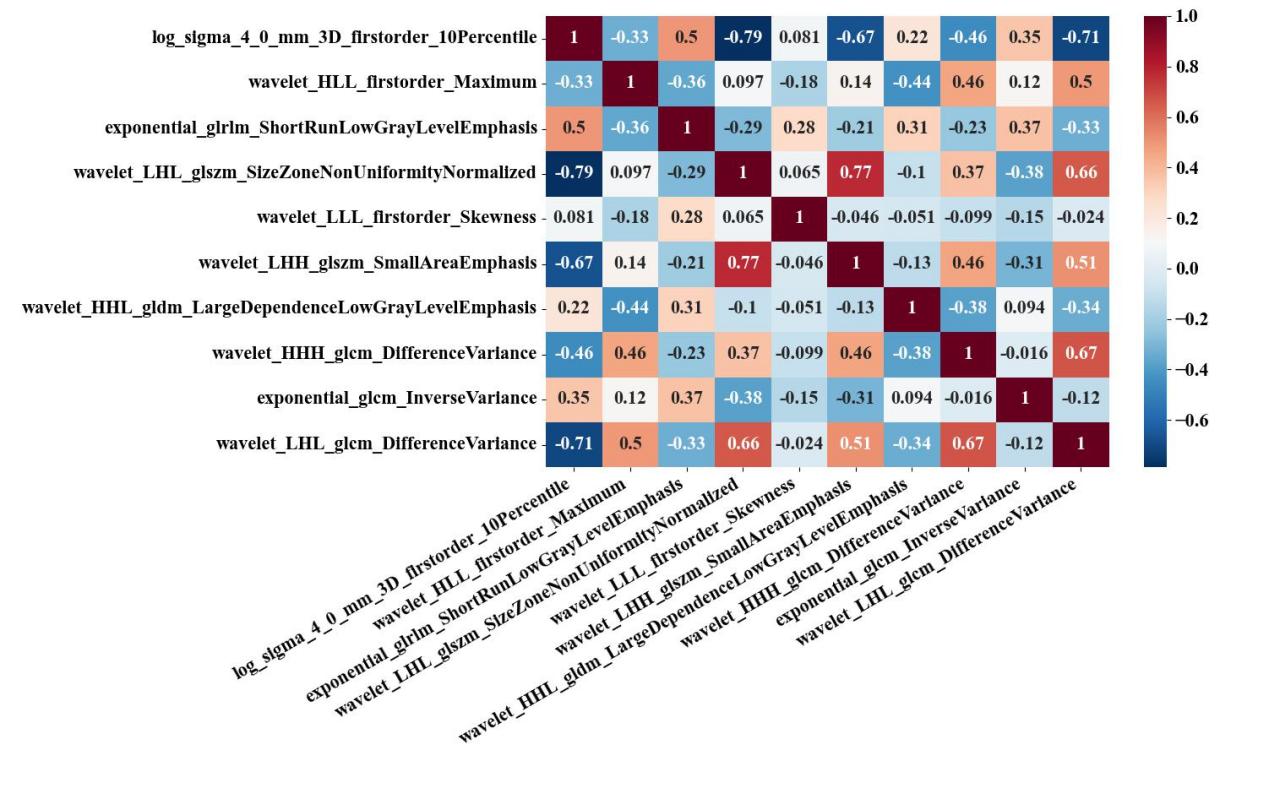
**

1. **DWI b0**

**
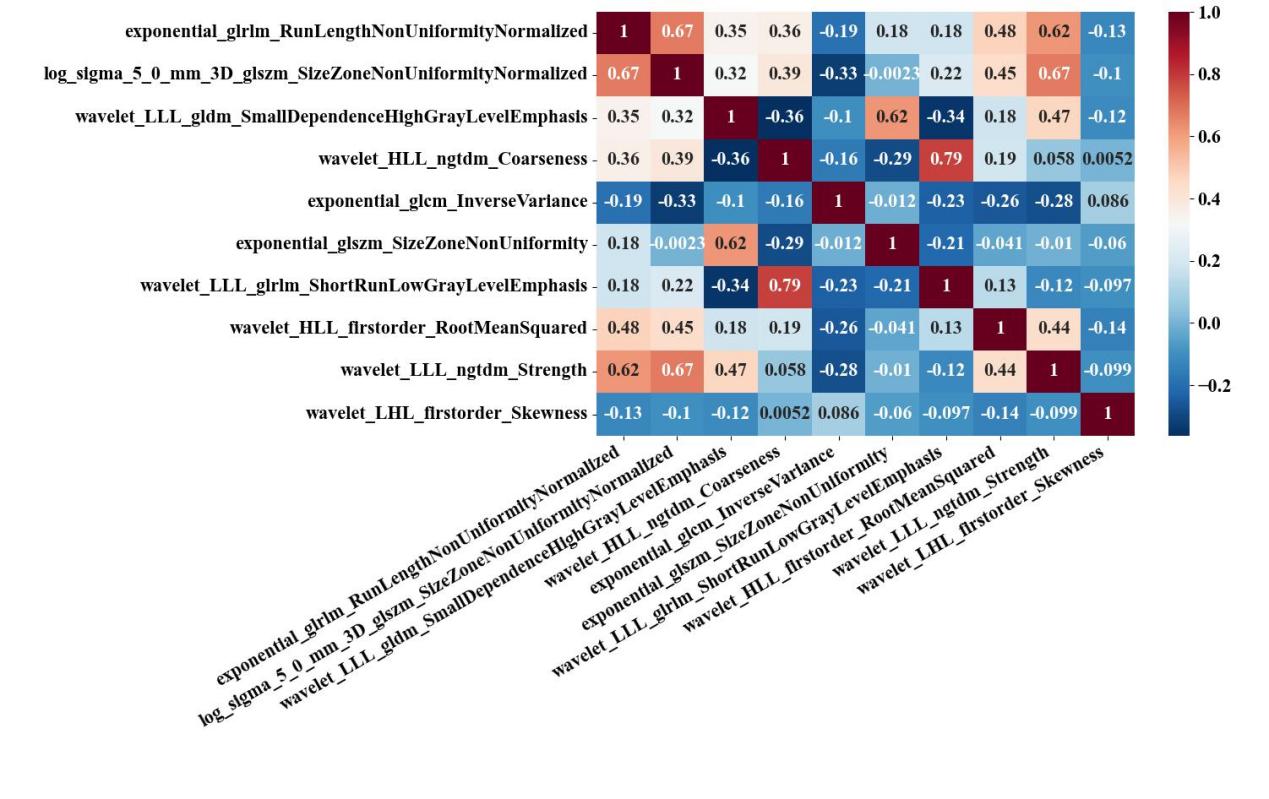
**

1. **DWI b50**

**
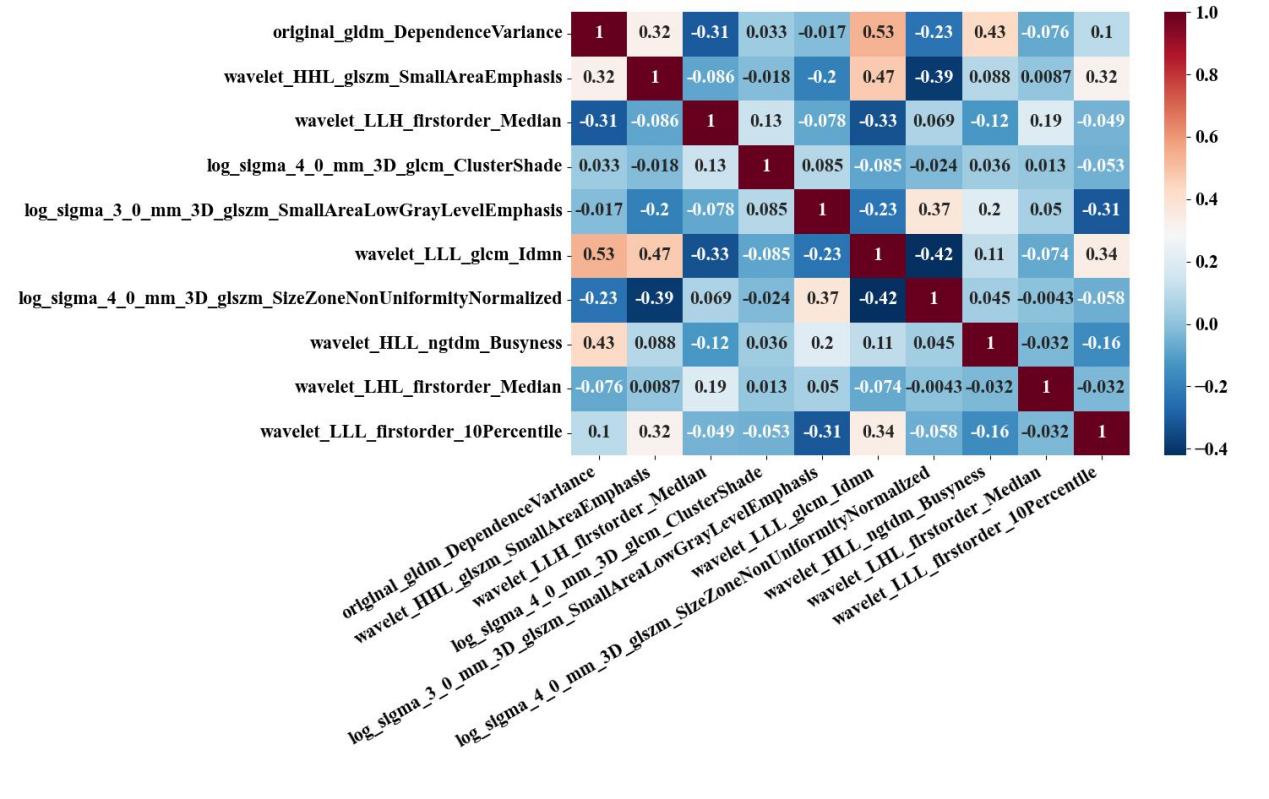
**

1. **DWI b800**

**
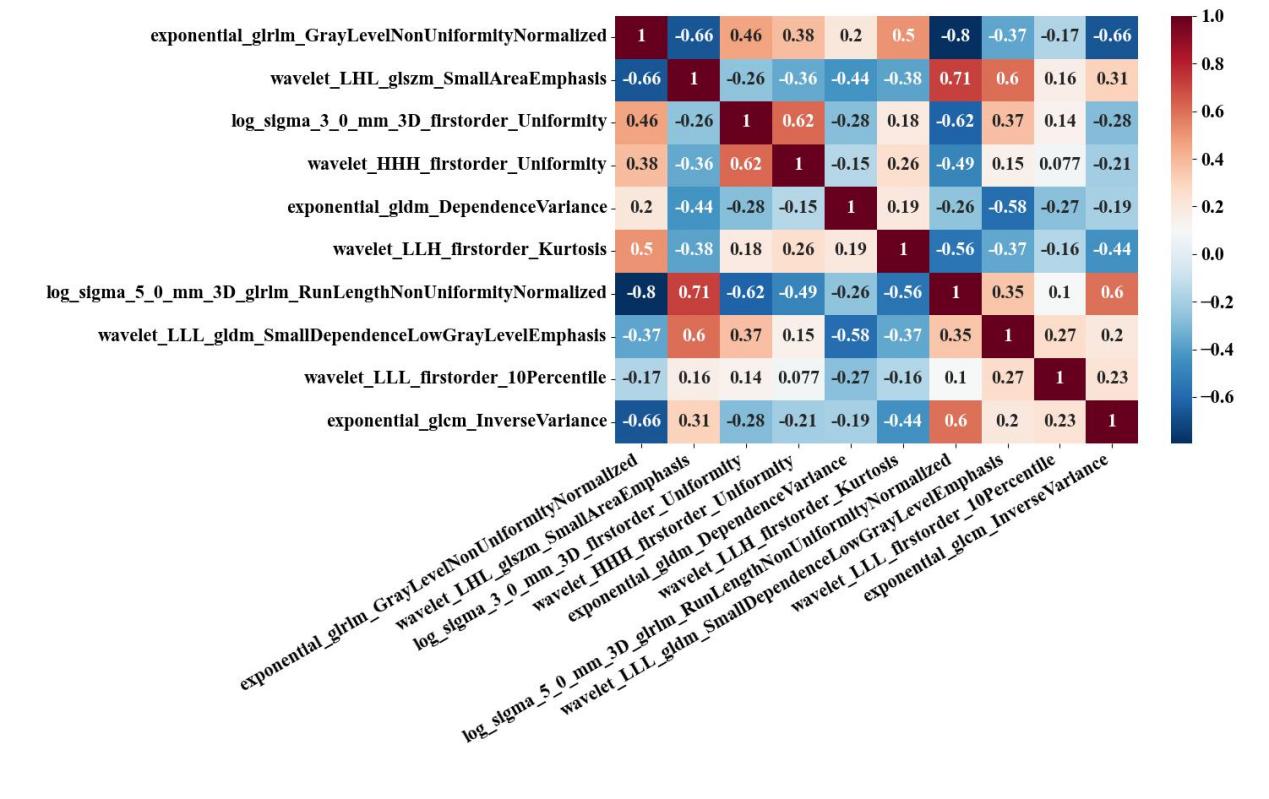
**

1. **Diffusion-derived vessel density**
